# Monoclonal antibodies that recognize important functional elements of the HIV-1 integrase enzyme

**DOI:** 10.1186/1742-4690-9-S1-P6

**Published:** 2012-05-25

**Authors:** Richard G Maroun, Farah Ammar, Zeina Hobaika, Loussinée Zargarian, Serge Fermandjian

**Affiliations:** 1 Unité de Biochimie, Faculté des Sciences, Université Saint-Joseph, CST-Mar Roukoz, Beirut, Lebanon; 2 ENS de Cachan, CNRS, Cachan, France

## Aim

HIV integrase (IN) is a privileged target for antiviral treatments. These induce the emergence of resistant strains, prompting the search of new drugs. To better understand the relationships between structure and function of IN and identify new anti-HIV inhibitors we prepared antibodies recognizing the IN a4 helix that binds viral DNA ends and contributes to the integration process and antibodies recognizing a loop in between the a4 and a5 helices which participates to the binding of LEDGF a protein that helps IN to anchor viral DNA.

## Materials and methods

Polypeptide K159 (sequence 147-175 of IN) was injected to mice. Several hybridomas producing monoclonal antibodies (Mabs) were obtained Mabs were characterized by ELISA and blotting techniques using peptide fragments, IN and viral DNA sequences.

## Results

We prepared two Mabs (Mab-a4 and Mab-loop) exhibiting high affinities against the antigenic peptide K159 and IN. An epitope mapping showed that Mab-a4 interacted with N-terminal segment (147-163) and Mab-loop with the C-terminal (164-175). Mab-a4 blocked the interaction of IN with viral DNA end, while the loop segment 164-175 recognized by the Mab-loop constitutes a strong epitope also found in African seropositive patients. Spectroscopic studies of the antibody-antigen complexes are under progress. Crystallization of the Fab moiety of Mab-a4 has been recently obtained.

## Conclusions

We showed that the important immunogenic properties demonstrated by the a4 helix and the loop 164-175 coincided with their important functional properties in IN. We wish to collect details on the interactions and the energies stabilizing these complexes and compare them with those stabilizing the complexes with their biological targets (DNA, LEDGF). Finally at a medical level, these Mab could be used as valuable tools for HIV diagnostics in ELISA or western blot assays.

